# Identification of residues within the African swine fever virus DP71L protein required for dephosphorylation of translation initiation factor eIF2α and inhibiting activation of pro-apoptotic CHOP

**DOI:** 10.1016/j.virol.2017.02.002

**Published:** 2017-04

**Authors:** Claire Barber, Chris Netherton, Lynnette Goatley, Alice Moon, Steve Goodbourn, Linda Dixon

**Affiliations:** a The Pirbright Institute, Ash Road, Pirbright, Woking, Surrey GU24 0NF, United Kingdom; b Institute for Infection and Immunity, St. George's, University of London, London SW17 0RE, United Kingdom

**Keywords:** EIF2α, Protein translation, CHOP, Unfolded protein response, African swine fever virus

## Abstract

The African swine fever virus DP71L protein recruits protein phosphatase 1 (PP1) to dephosphorylate the translation initiation factor 2α (eIF2α) and avoid shut-off of global protein synthesis and downstream activation of the pro-apoptotic factor CHOP. Residues V16 and F18A were critical for binding of DP71L to PP1. Mutation of this PP1 binding motif or deletion of residues between 52 and 66 reduced the ability of DP71L to cause dephosphorylation of eIF2α and inhibit CHOP induction. The residues LSAVL, between 57 and 61, were also required. PP1 was co-precipitated with wild type DP71L and the mutant lacking residues 52- 66 or the LSAVL motif, but not with the PP1 binding motif mutant. The residues in the LSAVL motif play a critical role in DP71L function but do not interfere with binding to PP1. Instead we propose these residues are important for DP71L binding to eIF2α.

## Highlights

• The African swine fever virus DP71L protein recruits protein phosphatase 1 (PP1) to dephosphorylate translation initiation factor eIF2α (eIF2α).
• The residues V^16^, F^18^ of DP71L are required for binding to the α, β and γ isoforms of PP1 and for DP71L function.
• The sequence LSAVL downstream from the PP1 binding site (residues 57–61) are also important for DP71L function.
• DP71L mutants of the LSAVL sequence retain ability to co-precipitate with PP1 showing these sequences have a different role to PP1 binding.

## 1 Introduction

Many viruses encode proteins that inhibit the shut-off of global protein synthesis mediated by the phosphorylation of the eukaryotic translation initiation factor 2α (eIF2α)⊡ Phosphorylation of eIF2α is carried out by protein kinases, including the double-stranded RNA activated protein kinase PKR and endoplasmic reticulum resident (ER) PERK protein kinase, which is activated as part of the cellular unfolded protein response (UPR). The UPR is activated following accumulation of unfolded or misfolded proteins within the ER. The three central mediators of the UPR; PERK, IRE1 and ATF6, work together to restore homeostasis within the ER. However, prolonged activation of the UPR can lead to apoptosis and autophagy (Bernales et al., 2006, Chakrabarti et al., 2011, Hetz, 2012).

Phosphorylation of translation initiation factor eIF2α on Serine 51, leads to attenuation of protein synthesis due to the increased affinity of eIF2α for the guanine nucleotide exchange factor, eIF2B. The inhibition of eIF2B by eIF2α limits the formation of the pre-initiation complex, which is required for translation initiation. Since eIF2B is present in rate-limiting quantities, small changes in the phosphorylation status of eIF2α can significantly affect translation initiation (Dever, 2002, Krishnamoorthy et al., 2001).

A small subset of stress-related proteins are still synthesised when eIF2α is phosphorylated. These include the transcription factor ATF4 and downstream targets including the pro-apoptotic transcription factor CHOP, C/EBP homologous protein (Harding et al., 2000, Harding et al., 2003). CHOP mediates the down-regulation of the anti-apoptotic protein Bcl2, depletes cellular glutathione and increases production of reactive oxygen species, sensitising the cell to ER stress and apoptosis (McCullough et al., 2001).

**Fig. 1:**
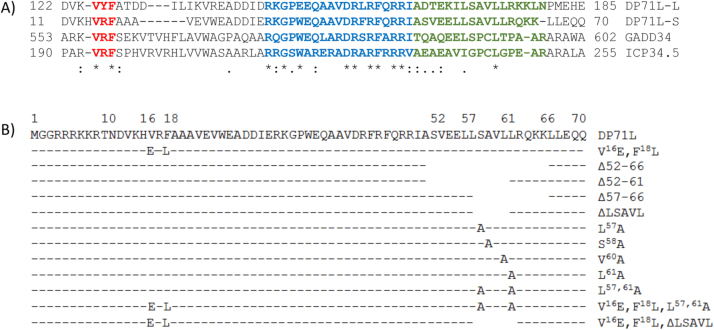
*Alignment of DP71L with domains from GADD34 and ICP34.5 and mutants of DP71L* Panel A) Shows an alignment of the long and short forms of DP71L with the C terminal domain of ICP34.5 of HSV-1 and GADD34. Within the C terminal region of ICP34.5 residues 233–248 (green) have been identified as the eIF2α binding domain (Li et al., 2011), whilst the eIF2α binding motif described by Rojas et al. (2015) in GADD34 is shown in blue. The predicted PP1 binding motif is highlighted in red. Panel B) shows the sequences of mutants of DP71L generated in this work. Numbers denote positions within wild type short DP71L sequences that correspond to mutations made. Dashed lines indicate the sequence is not altered from the wild type sequence and gaps show sequences deleted.

African swine fever virus (ASFV), is a large cytoplasmic DNA virus which encodes over 150 proteins, (Dixon et al., 2005). ASFV encodes DP71L protein, which acts like the host GADD34 (growth arrest and DNA damage-inducible protein 34) and Herpes simplex virus ICP34.5 protein to recruit PP1, to dephosphorylate eIF2α and so restore protein synthesis (Brush et al., 2003; Li et al., 2011; Novoa et al., 2001; Zhang et al., 2008, Zhang et al., 2010). ASFV isolates encode either a long (184 amino acids) or short form 70–72 amino acids) of DP71L. These share a conserved C-terminal domain with ICP34.5 and GADD34. The additional N-terminal domain of the long form does not share similarity with other proteins (Alonso et al., 2013; Rivera et al., 2007). ICP34.5 and GADD34 interact directly with PP1 via a canonical binding domain, although this interaction alone is not sufficient for the dephosphorylation of eIF2α (He et al., 1997). Residues 233–248 of ICP34.5 have been described as containing an eIF2α binding domain (Li et al., 2011), although the critical interacting residues within this domain have not been further characterised. The C-terminal domains of DP71L, ICP34.5 and GADD34 share conserved residues including the PP1 binding domain (see Fig. 1). Previously (Zhang et al., 2010) we demonstrated that phosphorylated eIF2α is undetectable in resting cells expressing DP71L, and this is not increased upon treatment with ER stress inducers. Interactions between the ASFV homologue DP71L, PP1 and eIF2α were demonstrated through co-immunoprecipitation and yeast three-hybrid experiments. We proposed that DP71L recruits PP1 to dephosphorylate eIF2a, however, the residues involved in these interactions have not yet been characterised (Rivera et al., 2007; Zhang et al., 2010). In this study we identified functionally critical residues in DP71L, including, a sequence downstream of the PP1 binding domain which we propose binds to eIF2α. The results provide insights into how DP71L acts and is of special interest since the 71 amino acid DP71L protein is the shortest known protein with this function.

## 2 Results

### 2.1 DP71L inhibits activation of CHOP induced by tunicamycin

DP71L expression has been shown to result in de-phosphorylation of eIF2α. This results in inhibition of the downstream induction of ATF4 and CHOP (Zhang et al., 2010). The ability of mutant forms of DP71L proteins to inhibit induction of CHOP was therefore used to identify functionally important residues in the protein. The conditions for activation of CHOP by tunicamycin in Vero cells were optimized, and 20 μg/ml tunicamycin treatment for 8 h shown to activate and induce nuclear localisation of CHOP (data not shown).

**Fig. 2:**
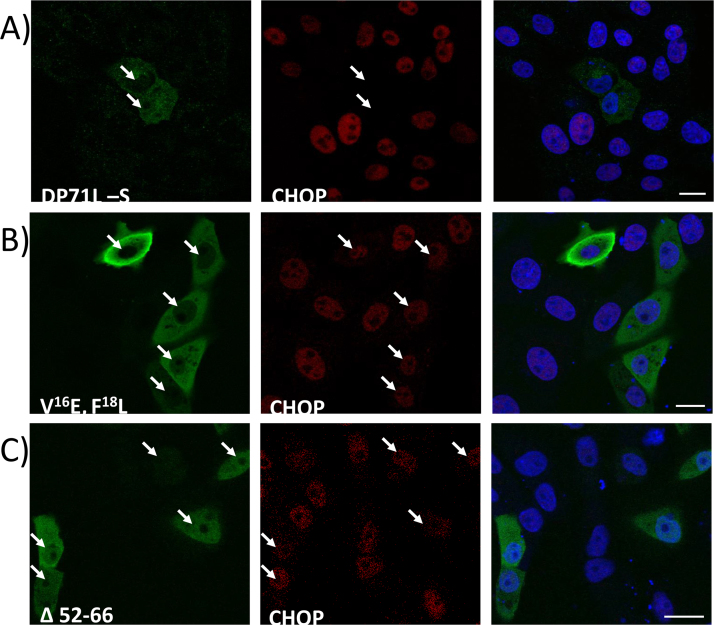
*The residues V*^*16*^*, F*^*18*^*and 52–67 are required for function of DP71L.* Vero cells were transfected with plasmids expressing HA epitope tagged wild type (A) or mutant DP71L, panel B, V^16^E, F^18^L or lacking residues 52–67 (panel C). At 24 h post-transfection cells were stimulated with 20 µg/ml tunicamycin for 8 h to induce expression of CHOP. Cells were then fixed in 4% PFA, permeabilised and labelled with DAPI, anti-HA and anti-CHOP antibodies. Primary antibodies were visualised with appropriate secondary reagents conjugated to Alexa 488 or Alexa 568 respectively. Arrows point to the nuclei of transfected cells. Scale bars represent 20 µm.

**Fig. 3:**
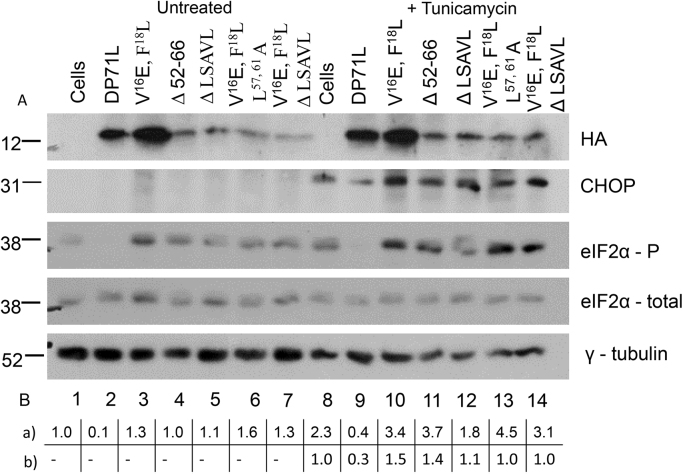
*Wild type, but not mutant DP71L causes dephosphorylate eIF2α* A) Vero cells were mock-transfected or transfected with wild type or mutant DP71L as indicated on the figure. At 24 h post-transfection cells were stimulated with 20 μg/ml tunicamycin for 8 h and then lysed. 20 μg of total protein from lysates was resolved by SDS-PAGE and transferred to membranes prior to blotting with antibodies against the HA epitope tag, phosphorylated and total eIF2α, CHOP and γ tubulin. The positions of molecular mass markers are indicated to the left of the gel (in Kilo Daltons). B) a) The relative level of phosphorylated to total eIF2α was determined by densitometry analysis using ImageJ software, and normalised to the ratio observed in lane 1. b) The relative ratio of CHOP to total eIF2α was determined as above, and expressed relative to the ratio observed in lane 8. The mean ratio was calculated from three independent experiments.

Mutants of the DP71L short form protein (DP71Ls) with an N-terminal HA epitope tag, were constructed and compared with the wild type protein for the ability to inhibit the induction and nuclear localisation of CHOP. Initially mutant genes were constructed that had the predicted PP1 binding site mutated (V^16^E, F^18^L, Fig. 1B). In addition deletions were made of C-terminal sequences. This region is similar in location, relative to the predicted PP1 binding site, to the putative eIF2α binding domain of ICP34.5 (residues 233–248 ICP34.5, Fig. 1A). Following transfection of plasmids expressing these mutant DP71L proteins into tunicamycin-treated or untreated cells, expression of CHOP was tested by confocal microscopy (Fig. 2) or by Western blotting of cell extracts (Fig. 3). A summary of results showing the level of CHOP induced following transfection of plasmids expressing different DP71L mutants into cells is shown in Table 1. Expression of DP71L protein was detected to varying levels following transfection of all tested plasmids tested (data not shown and Fig. 3). No expression was detected from plasmids that encoded DP71L proteins with the C-terminal 10 or 20 amino acids deleted (data not shown).

Fig. 2 shows representative confocal images for cells expressing HA-tagged wild type (A) and mutant DP71L proteins including mutants of residues V^16^E, F^18^L (B) and deletion of residues 52–66 (C). The presence of CHOP within the nucleus of 150–200 transfected cells expressing these mutant proteins was assessed visually. Mutation of the residues V^16^E, F^18^L or deletion of residues 52–66 (Δ 52–66) resulted in increased numbers of cells expressing CHOP within the nucleus compared to cells expressing the wild type protein. Cells expressing mutants DP71L V^16^E, F^18^L and DP71L Δ 52–66 demonstrated inhibition of CHOP nuclear localisation in 17% and 13% of cells compared to 98% in wild type DP71L expressing cells (Fig. 3 panel B and C compared to A). The results suggest the residues V^16^, F^18^ and residues 52–66 are required for the inhibition of CHOP induction by DP71L.

To better define the regions within the residues 52–66 that are required to inhibit CHOP activation deletions were made of residues 52–61 (−20 to −10 from the C-terminus) and 57–66 (−15 to −5 from the C-terminus) (see Fig. 1). A reduction in the efficiency of CHOP inhibition from 98% in cells expressing wild type DP71L to 23% and 18% in cells expressing DP71L Δ 52–61 and DP71L Δ 57–66 was observed respectively (Table 1). These mutants lack a common motif (LSAVL) between residues 57–61. A mutant, DP71L Δ LSAVL, lacking these residues also had a reduced ability to inhibit the accumulation of CHOP within the nucleus when compared to wild type DP71L (39% compared to 98% CHOP inhibition) (Table 1).

### 2.2 The two leucine residues within the LSAVL motif are most important for DP71L inhibition of CHOP nuclear localisation

Each residue within the LSAVL motif was replaced with an alanine. Mutating residues S^58^ and V^60^ had no effect on the wild type function of DP71L as CHOP was detected in the nucleus of transfected cells at similarly high levels as in cells expressing wild type DP71L (Table 1). Mutation of the two leucine residues to alanine reduced the ability of DP71L to inhibit CHOP nuclear localisation (Table 1). Of these two leucine residues mutation of L^57^ had the greater effect, reducing the percentage of CHOP inhibition to 31%, compared to 57% for mutant DP71L L^61^A, compared to 98% for wild type DP71L (Table 1). Together, these results suggest that the PP1 binding domain and the motif LSAVL are critical for function, and of the LSAVL motif the two leucine residues are the most important. DP71L proteins with these domains mutated retain some ability to inhibit CHOP induction suggesting additional residues may be important for activity or that the substitutions did not completely inactivate the protein. Mutants with both the mutation V^16^E, F^18^L and deletion of LSAVL domain were generated (V^16^E, F^18^L ΔLSAVL) and tested for their ability to inhibit CHOP activation. Mutant DP71L L^57, 61^A has both the leucine residues within the LSAVL motif mutated to alanine, whilst mutant DP71L V^16^E, F^18^L, L^57, 61^A lacks both leucine residues and has mutation V^16^E, F^18^L. Finally, mutant DP71L V^16^E, F^18^L, Δ LSAVL has the mutation V^16^E, F^18^L, and deletion of the LSAVL motif.

These mutants had reduced ability 20–31% compared to 98%) to inhibit CHOP nuclear localisation compared to the wild type DP71L (see Table 1). This suggests that although the V^16^,F^18^ residues and LSAVL sequence have important roles, there are additional residues required for the ability of DP71L to inhibit CHOP induction.

### 2.3 Expression of wild type DP71L but not forms mutated in the V^16^,F^18^ residues or LSAVL motif results in dephosphorylation of eIF2α

The inhibition of nuclear localisation of CHOP by wild type DP71L in cells undergoing ER stress is predicted to be a downstream effect of the DP71L mediated recruitment of PP1 to dephosphorylate eIF2α. Therefore, we tested for levels of dephosphorylated eIF2α in cells transfected with plasmids expressing mutant DP71L proteins.

Plasmids expressing the selected DP71L mutants were transfected into cells which were treated with tunicamycin, or left untreated. Western blots of cell lysates were probed with antibodies that recognised total or phosphorylated eIF2α (38 kDa), CHOP (30 kDa), the HA epitope-tagged DP71L protein and the loading control γ tubulin (51 kDa) (see Fig. 3). The levels of expression of wild type DP71L and the V^16^E,F^18^L mutant was consistently higher than that of other mutants. The mean relative ratio of phosphorylated eIF2α to total eIF2α and of CHOP to total eIF2α was determined relative to control Vero cells (see Fig. 3 panel B) from three independent experiments.

In all untreated cells CHOP was not detected but was induced by tunicamycin treatment. In control cells, as expected, tunicamycin treatment increased the level of eIF2α phosphorylation compared to untreated cells. In contrast in cells expressing wild type DP71L the band corresponding to phosphorylated eIF2α was not detected in either untreated or tunicamycin-treated cells (Fig. 3 lanes 2 and 9) although the total level of eIF2α remained stable (compare Fig. 3 lanes 1 and 8). In tunicamycin-treated cells a band corresponding to the CHOP protein was detected, although reduced in amount relative to that observed in the untransfected control cells, with a mean relative ratio of 0.3 compared to 1. In lysates from cells expressing DP71L which had the V^16^E, F^18^L mutation a higher level of eIF2α phosphorylation was detected in both untreated and tunicamycin-treated cells with relative ratios of 1.3 and 3.4 respectively compared to 1 in control Vero cells (compare Fig. 3 panel A lanes 3 and 10, and panel B). In these lysates CHOP was strongly induced following tunicamycin treatment (Fig. 3 lane 10). In lysates from cells expressing DP71L Δ52–66 phosphorylated eIF2α was detected in both resting and tunicamycin-treated cells, indicating that this mutant no longer caused dephosphorylation of eIF2α. Furthermore, the CHOP protein was expressed upon stimulation with tunicamycin (Fig. 3 lane 11).

In cells expressing DP71L with mutations in V^16^E, F^18^L and the LSAVL motif the following results were obtained. In lysates of cells expressing DP71L with a deletion ΔLSAVL, this deletion in combination with V^16^E, F^18^L, Δ LSAVL, and V^16^E, F^18^L, L^57, 61^A phosphorylated eIF2α was detected in untreated cells, and increased following tunicamycin treatment (compare Fig. 3 lanes 5–7 and 12–14). The CHOP protein was also detected in tunicamycin-treated cells expressing DP71L mutants with the LSAVL sequence deleted (ΔLSAVL), with V^16^E, F^18^L, mutated in combination with the LSAVL sequence mutated (V^16^E, F^18^L, L^57, 61^A) and with the mutated in combination with the LSAVL sequence deleted (V^16^E, F^18^L, ΔLSAVL).

Interestingly, lysates from cells expressing DP71L Δ52–66 had the highest relative ratio between phosphorylated and total eIF2α in the untreated cell lysates (Fig. 3 A lane 4, 1.8 to 1) and was also higher in the tunicamycin-treated cells (Fig. 3 A lane 11, 3.6 to 2.3) compared to other mutants tested. This mutant form of DP71L may still bind PP1 and thus sequester PP1 preventing its interaction with cellular factors which control the level of eIF2α phosphorylation. This could explain the observed increase in the level of phosphorylated eIF2α in cells expressing this DP71L mutant.

The data confirmed that mutation of the V^16^, F^18^ residues or LSAVL motif in DP71L resulted in the loss of wild type DP71L ability to cause dephosphorylation of eIF2α and inhibit induction of CHOP following tunicamycin treatment.

### 2.4 DP71L mutants with mutations in residues V^16^, F^18^ do not co-precipitate with PP1

**Fig. 4:**
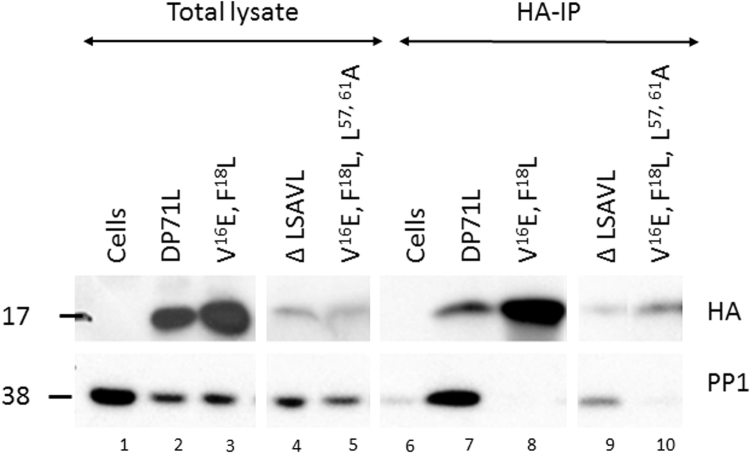
*DP71L mutant V16E, F18L does not co-precipitate with PP1* Vero cells were transfected with plasmids expressing wild type or mutant DP71L, 24 h post-transfection lysates were harvested and incubated overnight with the HA affinity matrix at 4 °C with rotation. Lysates were pelleted and washed three times prior to re-suspension in SDS-PAGE loading buffer. Samples were resolved by SDS-PAGE, transferred to membranes by Western blot and probed against the HA epitope tag and PP1.

Our previous results using the yeast three hybrid system indicated that DP71L binds to PP1 and this complex interacts with eIF2α (Zhang et al., 2010). A prediction from this is that mutation of the PP1 binding domain in DP71L would reduce the interaction between these proteins and the failure to recruit PP1 to eIF2α would explain the failure to inhibit eIF2α phosphorylation. Levels of the wild type and mutant DP71L detected in total lysates detected by Western blotting varied (Fig. 3). The wild type DP71L, V^16^E, F^18^L, and this mutation combined with a deletion of the LSAVL sequence (V^16^E, F^18^L, ΔLSAVL) were expressed at highest levels. Anti-HA was used to precipitate DP71L and interacting proteins from cell lysates. The co-precipitates were resolved by SDS/PAGE and Western blotting carried out with antibodies to PP1 and anti-HA (Fig. 4). As expected, PP1 was strongly co-precipitated from cells expressing wild type DP71L (Fig. 4. lane 8). PP1 was not co-precipitated with the mutant DP71L V^16^E, F^18^L, indicating that these residues are critical for the interaction between DP71L and PP1 (Fig. 4, lane 9). PP1 was co-precipitated with mutant DP71L Δ LSAVL (Fig. 4 lane 10). Thus the ΔLSAVL mutants still co-precipitated with PP1 indicating these residues are not critical for this interaction.

**Fig. 5:**
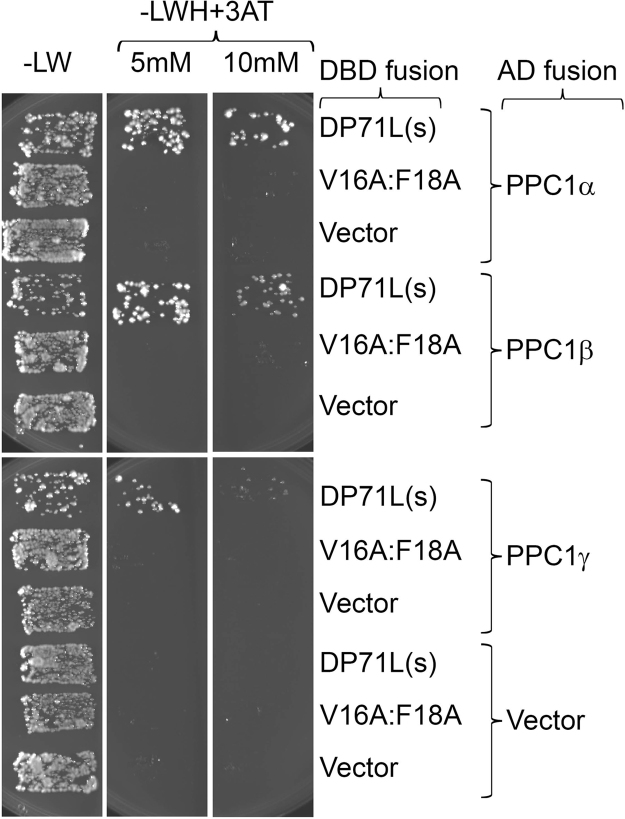
*The V16E; F18L form of DP71L is unable to interact with protein phosphatase isoforms* Panel B. Yeast strain PJ69-4α was transformed with pairs of plasmids expressing the indicated DNA binding hybrid and the phosphatase (PPC1) isoform fused to the yeast GAL4 activation domain. Yeast containing both plasmids were selected on synthetic drop-out medium lacking leucine and tryptophan (“-LW”) and then streaked onto synthetic drop-out medium lacking leucine, tryptophan and histidine and containing 5mJM or 10 mM 3-aminotriazole (“-LWH +3AT”). Growth on the latter is indicative of protein-protein interaction.

The interaction of wild type DP71L and the PP1 binding mutant DP71L V16A, F18L with all three isoforms of PP1 was investigated using the yeast two-hybrid system. As shown in Fig. 5 the wild type DP71L interacted strongly with the α, and β ιsoforms of PP1, and also interacted with the γ isoform (albeit more weakly, since the interaction was weak in the presence of 10 mM 3-aminotriazole). In contrast the DP71L V^16^E, F^18^A did not bind to any of the isoforms even at low stringency.

### 2.5 Wild type, but not mutant, DP71L acts as a translation enhancer

To assess whether the DP71L mutants which had reduced ability to dephosphorylate eIF2α had also lost their ability to act as translation enhancers, a bi-cistronic reporter plasmid was used with the firefly luciferase gene downstream of the CMV promoter and the renilla luciferase gene downstream of the ECMV IRES. Using this reporter plasmid, both cap-dependent and independent translation initiation can be assessed by measuring levels of firefly and renilla luciferase respectively. It is predicted that DP71L would enhance both cap dependent and independent translation, as eIF2α is required for translation initiation of both.

**Fig. 6:**
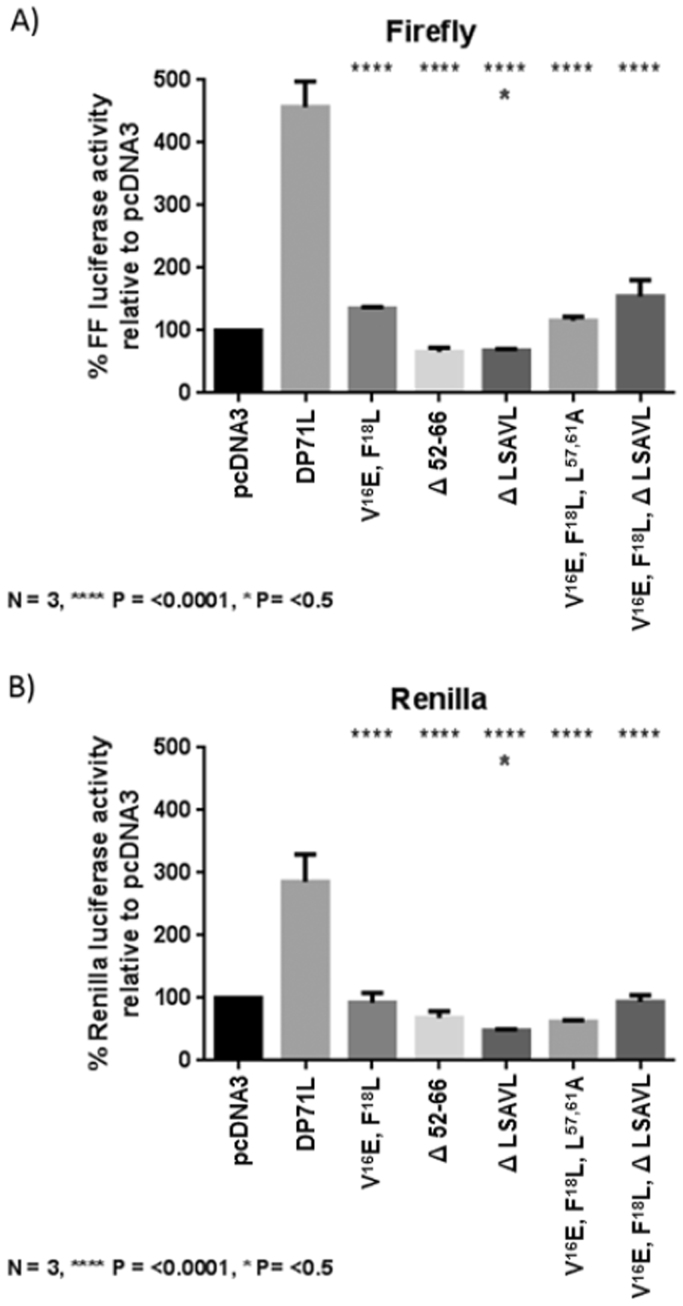
*Wild type, but not mutant, DP71L acts as a translation enhancer* Vero cells were co-transfected with equal amounts of the bi-cistronic reporter plasmid pIRES FF luc/Ren luc and pcDNA3, wild type or mutant DP71L as indicated. 24 h post-transfection cells were lysed and reporter activity assessed using the Dual-Luciferase Reporter Assay kit (Promega). The firefly (A) or renilla (B) reporter activity of control cells transfected with pcDNA3 was set at 100% and wild type or mutant activity expressed as a percentage relative to pcDNA3. Experiments were performed in triplicate three times. Error bars represent the standard deviation. Statistical analysis was carried out in GraphPad Prism using a one way ANOVA with multiple comparisons test. Asterisks represent a significant difference in value between WT DP71L and the mutants tested (*= *P* value of <0.5, ****= *P* value of <0.0001).

Vero cells were co-transfected with equal amounts of the bi-cistronic reporter plasmid and pcDNA3 expressing wild type or mutant DP71L or empty vector. The reporter activity of control cells transfected with pcDNA3 was set at 100% and the activity in cells transfected with wild type or mutant DP71L expressed as a percentage relative to pcDNA3. Fig. 6 shows that DP71L efficiently enhanced translation of both cap-dependent and independent translation by over 450% of the pcDNA3 control for firefly luciferase cap dependent translation (panel A), and just under 300% for renilla luciferase cap independent translation (panel B). In contrast, all of the DP71L mutants tested abolished this enhancement, as the levels of reporter expression were similar to the plasmid control. By performing a one way ANOVA in GraphPad Prism with multiple comparisons test against wild type DP71L, it was established that each of the mutants significantly reduced the translation enhancer effect of DP71L (*P*≤0.0001).

Plasmids expressing mutants DP71L Δ52-66 and DP71L Δ LSAVL consistently displayed a reduced induction of reporter activity compared to the pcDNA3 control plasmid, and this reached statistical significance for the DP71L mutant Δ LSAVL, (*P* value of <0.05 see Fig. 6, blue asterisk). Possibly because these DP71L mutants still bind PP1 they may sequester PP1 from cellular factors such as CReP, reducing de-phosphorylation of eIF2α, and so decreasing translation below basal conditions.

## 3 Discussion

Shut-off of host protein synthesis is a major limitation to viral replication, and as such many viruses, including ASFV, have evolved mechanisms to evade or limit this response. The DP71L protein acts by targeting the cellular phosphatase PP1 to dephosphorylate translation initiation factor eIF2α to avoid the shut-off of global protein synthesis. This may be induced by either the double-stranded RNA activated protein kinase PKR or the protein kinase-like ER resident kinase (PERK), which is activated as part of the unfolded protein response (UPR). The UPR also feeds into the innate immune response through the activation of pro-inflammatory cytokines and NF-κB. This occurs via the IRE1/XBP1 and PERK pathways, as for example, IL-6 and IL-8 are targets of XBP1, whilst the shut off of protein synthesis by PERK leads to an imbalance in the ratio of IκB to NF-κB, leading to NF-κB activation (Deng et al., 2004, Gargalovic et al., 2006, Kaneko et al., 2003, Urano et al., 2000). In previous studies ASFV infection of Vero cells was shown to activate the ATF6 branch (ATF6 activates ER chaperones and XBP1) of the UPR but not PERK or the inositol-requiring enzyme (IRE1) (Galindo et al., 2012, Netherton et al., 2004). This evidence suggests that ASFV infection does not cause PERK activation and thus DP71L function may primarily function to counteract PKR-activated phosphorylation of eIF2α.

In this study we identified critical residues which are required for function of DP71L to reduce phosphorylation of translation initiation factor eIF2α and to inhibit the downstream effects including induction of the pro-apoptotic CHOP protein in response to stress induced by tunicamycin. We confirmed that the residues V^16^, F^18^ within a predicted PP1 binding site (VRF) in DP71L were required for these functions. We showed that wild type DP71L co-precipitated with PP1 whereas DP71L with a mutation V^16^E, F^18^L did not. Thus we conclude that these residues are essential for PP1 binding and function of DP71L.

We expected to identify a domain in DP71L required for binding to eIF2α and investigated whether sequences downstream from the PP1 binding domain were required for DP71L function. Mutation of the sequence LSAVL between residues 57 and 61 reduced DP71L ability to cause dephosphorylation of eIF2α and inhibit CHOP induction. Within this LSAVL sequence the two leucine residues were most critical. DP71L mutants of the LSAVL sequence retained the ability to co-precipitate PP1 suggesting that the LSAVL sequence has a critical functional role other than PP1 binding. We failed to detect co-precipitation of eIF2α with DP71L and PP1, possibly due to a weak or transitory interaction (data not shown). Therefore we were unable to confirm that this sequence is involved in binding of DP71L to eIF2α although consider this likely.

Studies with ICP34.5 and GADD34 proteins have also investigated domains critical for function. In one study (Li et al., 2011) the eIF2α binding domain of ICP34.5 was mapped to residues 233–248; and in GADD34 the sequence between residues 578–597, Rx[Gnl]x1-2Wxxx[Arlv]x[Dn][Rg]xRFxx[Rlvk][Ivc] (where capital letters are preferred and x is any residue) was described (Rojas et al., 2015) as the eIF2α binding (see Fig. 1). Further investigation of residues in this sequence critical for DP71L function would be of interest. In ICP34.5 and GADD34 an additional RARA motif has been implicated in stabilising PP1 binding (Brush et al., 2003, Zhang et al., 2008). A similar sequence is not present in either the long form or short form of DP71L.

It is interesting that the leucine residues within the DP71L LSAVL sequence are highly conserved between ICP34.5, GADD34 and DP71L supporting the hypothesis that these amino acids are indeed involved in an interaction with eIF2α. Structure prediction analysis also suggests that these residues are highly exposed.

## 4 Materials and methods

### 4.1 Plasmids

Wild type or mutant DP71L genes were synthesised with an HA-tag of sequence YPYDVPDYA (Eurofins, London, UK) fused at the 5′ end of the gene. Genes were subsequently subcloned into the pEF-plink2 vector using the *Nco* I and *Xba* I restriction sites, and into pcDNA3 using the *Hind* III and *Bam H*I restriction sites. The plasmid pIRES FF luc/Ren luc was generated by inserting the firefly luciferase gene between the *Xho* I and *Mlu* I restriction sites, and the renilla luciferase gene between *Xba* I and *Not* I restriction sites of pIRES Neo.

### 4.2 Cell culture and transfection

Vero cells were maintained in Dulbecco's modified Eagle's medium (DMEM) containing 10% foetal bovine serum, penicillin (100 IU/ml) and streptomycin (100 μg/ml). Cells were seeded at 2.5×10^4^ cells/cm^2^ 16 h prior to transfection or experimental treatment. Transfections were performed in accordance with the TransIT-LT1 transfection reagent protocol (Mirus, USA). When required, cells were treated with tunicamycin, by replacing the culture media with media containing 20 μg/ml tunicamycin for 8 h, prior to harvesting for assay.

### 4.3 Luciferase assays

Lysates were prepared from cells that had been co-transfected with the reporter plasmid pIRES FF luc/Ren luc and control plasmids, or plasmids expressing either wild type or mutant DP71L. Lysates were analysed for luciferase activity using the dual luciferase reporter assay kit (Promega, UK), in accordance with the manufacturer's instructions.

### 4.4 Antibodies

Antibodies used included a mouse monoclonal against CHOP (B3, Santa Cruz, USA) used at a 1:200 dilution for confocal microscopy and Western blotting, a goat monoclonal against total eIF2α (K17, Santa Cruz) and a rabbit monoclonal against phosphorylated eIF2α (E90, Abcam, UK) used at 1:1000 and 1:500 for Western blotting respectively. The rat anti-HA antibody was from Roche (clone 3F10) and was used at a 1:500 dilution for confocal microscopy, and 1:1000 for Western blot. The goat polyclonal antibody against PP1 was acquired from Santa Cruz, USA (clone C19) and used at a dilution of 1:1000 for Western blot. The γ tubulin antibody (Sigma-Aldrich, T6557) was used at a 1:25,000 dilution for Western blot.

The following horseradish peroxidase (HRP) conjugated secondary antibodies were used for Western blotting at a 1:1000 dilution and were acquired from Dako, UK; rabbit anti-rat HRP (P0450), rabbit anti-mouse HRP (P0260), goat anti-rabbit HRP (P0448). In addition, the bovine anti-goat HRP antibody (Santa Cruz, USA sc-2384) was used at a 1:5000 dilution. The HA-HRP conjugated antibody (Roche, 3F10) was used at a 1:1000 dilution. Alexa Fluor antibodies goat anti-rat 488 and rabbit anti-mouse 568 were used at a 1:500 dilution for confocal microscopy.

### 4.5 SDS-PAGE and Western blotting

Cell lysates were harvested in RIPA buffer (Sigma) containing protease inhibitors (Sigma). The protein content was determined using the Pierce™ BCA Protein Assay Kit (Thermo-Fisher Scientific) and subsequently normalised, such that 10 or 20 µg of total protein could be loaded, per lane, onto SDS-PAGE gels. Proteins were resolved by SDS-PAGE and then transferred to Hybond PVDF membranes (GE Healthcare). Membranes were incubated for 1 h in PBS, 0.2% Tween-20, 5% skimmed milk powder, prior to an overnight incubation with the primary antibody diluted in the same blocking buffer. Membranes were washed in PBS containing 0.2% Tween-20, before incubating for 1 h with the HRP-conjugated secondary antibody. Membranes were washed prior to detection of chemiluminescence by incubating the membrane with LumiGLO (Cell Signalling Technology) for 1 min. Membranes were then exposed to X-ray film (Fujifilm) and developed manually using developer and fixer from AGFA.

### 4.6 Confocal microscopy

Cells were seeded onto glass coverslips (VWR) prior to transfection or other relevant treatment. At the experimental end-point, cells were washed with PBS (Gibco Thermo-Fisher Scientific), and fixed with 4% paraformaldehyde (PFA) for 1 h. Cells were permeabilised by incubation with PBS +0.1% Triton X-100 (Sigma-Aldrich) for 15 min, followed by blocking for 30 min with 0.5% BSA (Sigma-Aldrich) prior to antibody staining for 1 h with primary antibodies. Following several washes, coverslips were incubated with secondary antibodies for a further hour. Coverslips were washed, and DNA counterstained with DAPI (4′, 6-Diamidino-2-Phenylindole, Dihydrochrolide) prior to mounting. Cells were visualised using a Leica confocal laser scanning microscope, and data analysed using LCS Lite or LASAF Lite (Leica Confocal Software). Merged images were created in Image J.

### 4.7 Co-immunoprecipitation

Cells expressing HA-tagged wild type or mutant DP71L, and control non-transfected cells, were lysed in Pierce Protein Biology, Thermo Fisher Scientific IP lysis buffer supplemented with protease inhibitors. The lysate was incubated with anti-HA affinity matrix (Roche) overnight with rotation. The matrix was washed three times followed by centrifugation prior to resuspending the matrix in lysis buffer adding 5 x SDS-PAGE loading buffer. Samples were then boiled for 2 min, and the proteins resolved by SDS-PAGE then transferred to PVDF membranes (GE Healthcare) before Western blotting using primary and secondary antibodies as described.

### 4.8 Yeast two-hybrid

Combinations of GAL4 DBD and GAL4AD fusion plasmids were introduced into *Saccharomyces cerevisiae* strain PJ69-4α as described in Zhang et al. (2010) and selected on synthetic dropout medium lacking leucine and tryptophan. Individual colonies were subsequently streaked onto medium also lacking histidine and containing 5 mM or 10 mM 3-aminotriazole. Growth was monitored for 7 days at 30 °C.

## Figures and Tables

**Table 1:** Effect of wild type and mutant forms of DP71L on CHOP induction. Plasmids expressing wild type or mutant forms of DP71L were transfected into Vero cells. At 24 h post-transfection cells were stimulated with 20 µg/ml tunicamycin for 8 h to induce expression of CHOP. Cells were then fixed in 4% PFA, permeabilised and labelled with DAPI, anti-HA and anti-CHOP antibodies. Confocal microscopy was used to visualise 150–200 cells expressing the DP71L proteins and determine the percentage of those in which nuclear localisation of CHOP was induced. The first column shows the DP71L wild type or mutant proteins tested. The second column indicates the numbers of transfected cells in which CHOP nuclear localisation was detected. The – symbol indicates none and + or ++ increasing numbers of cells with CHOP detected in the nucleus. The third column expresses the percentages of transfected cells in which CHOP was detected in the nucleus.

	CHOP Induction	% CHOP inhibition
DP71Ls	–	98
V^16^E, F^18^L	++	17
Δ52–66	++	13
Δ52–61	++	23
Δ57–66	++	18
ΔLSAVL	+	39
L^57^A	+	31
S^58^A	–	98
V^60^A	–	98
L^61^A	+/-	57
L^57,61^A	+	25
V^16^E, F^18^L, L^57,61^A	++	20
V^16^E, F^18^L, ΔLSAVL	+	31

## References

[bib1] Alonso C., Galindo I., Cuesta-Geijo M.A., Cabezas M., Hernaez B., Muñoz-Moreno R. (2013). African swine fever virus-cell interactions: from virus entry to cell survival. Virus Res..

[bib2] Bernales S., McDonald K.L., Walter P. (2006). Autophagy counterbalances endoplasmic reticulum expansion during the unfolded protein response. Plos Biol..

[bib3] Brush M.H., Weiser D.C., Shenolikar S. (2003). Growth arrest and DNA damage-inducible protein GADD34 targets protein phosphatase 1 alpha to the endoplasmic reticulum and promotes dephosphorylation of the alpha subunit of eukaryotic translation initiation factor 2. Mol. Cell. Biol..

[bib4] Chakrabarti A., Chen A.W., Varner J.D. (2011). A review of the mammalian unfolded protein response. Biotechnol. Bioeng..

[bib5] Deng J., Lu P.D., Zhang Y.H., Scheuner D., Kaufman R.J., Sonenberg N., Harding H.P., Ron D. (2004). Translational repression mediates activation of nuclear factor kappa B by phosphorylated translation initiation factor 2. Mol. Cell. Biol..

[bib6] Dever T.E. (2002). Gene-specific regulation by general translation factors. Cell.

[bib7] Dixon L.K., Escribano J.M., Martins C., Rock D.L., Salas M.L., Wilkinson P.J., Fauquet C.M., Mayo M.A., Maniloff J., Desselberger U., Ball L.A. (2005). Asfarviridae., 2005. Asfarviridae. Virus Taxonomy, VIIIth Report of the ICTV.

[bib8] Galindo I., Hernaez B., Munoz-Moreno R., Cuesta-Geijo M.A., Dalmau-Mena I., Alonso C. (2012). The ATF6 branch of unfolded protein response and apoptosis are activated to promote African swine fever virus infection. Cell Death Dis..

[bib9] Gargalovic P.S., Gharavi N.M., Clark M.J., Pagnon J., Yang W.-P., He A., Truong A., Baruch-Oren T., Berliner J.A., Kirchgessner T.G., Lusis A.J. (2006). The unfolded protein response is an important regulator of inflammatory genes in endothelial cells. Arterioscler. Thromb. Vasc. Biol..

[bib10] Harding H.P., Novoa I., Zhang Y.H., Zeng H.Q., Wek R., Schapira M., Ron D. (2000). Regulated translation initiation controls stress-induced gene expression in mammalian cells. Mol. Cell.

[bib11] Harding H.P., Zhang Y.H., Zeng H.Q., Novoa I., Lu P.D., Calfon M., Sadri N., Yun C., Popko B., Paules R., Stojdl D.F., Bell J.C., Hettmann T., Leiden J.M., Ron D. (2003). An integrated stress response regulates amino acid metabolism and resistance to oxidative stress. Mol. Cell.

[bib12] He B., Gross M., Roizman B. (1997). The gamma(1)34.5 protein of herpes simplex virus I complexes with protein phosphatase 1 alpha to dephosphorylate the alpha subunit of the eukaryotic translation initiation factor 2 and preclude the shutoff of protein synthesis by double-stranded RNA-activated protein kinase. Proc. Natl. Acad. Sci. USA.

[bib13] Hetz C. (2012). The unfolded protein response: controlling cell fate decisions under ER stress and beyond. Nat. Rev. Mol. Cell Biol..

[bib14] Kaneko M., Niinuma Y., Nomura Y. (2003). Activation signal of nuclear factor-kappa B in response to endoplasmic reticulum stress is transduced via IRE1 and tumor necrosis factor receptor-associated factor 2. Biol. Pharm. Bull..

[bib15] Krishnamoorthy T., Pavitt G.D., Zhang F., Dever T.E., Hinnebusch A.G. (2001). Tight binding of the phosphorylated alpha subunit of initiation factor 2 (eIF2 alpha) to the regulatory subunits of guanine nucleotide exchange factor eIF2B is required for inhibition of translation initiation. Mol. Cell. Biol..

[bib16] Li Y., Zhang C., Chen X., Yu J., Wang Y., Yang Y., Du M., Jin H., Ma Y., He B., Cao Y. (2011). ICP34.5 protein of Herpes simplex virus facilitates the initiation of protein translation by bridging eukaryotic initiation factor 2 alpha (eIF2 alpha) and protein phosphatase 1. J. Biol. Chem..

[bib17] McCullough K.D., Martindale J.L., Klotz L.O., Aw T.Y., Holbrook N.J. (2001). Gadd153 sensitizes cells to endoplasmic reticulum stress by down-regulating Bc12 and perturbing the cellular redox state. Mol. Cell. Biol..

[bib18] Netherton C.L., Parsley J.C., Wileman T. (2004). African swine fever virus inhibits induction of the tress-induced proapoptotic transcription factor CHOP/GADD153. J. Virol..

[bib19] Novoa I., Zeng H.Q., Harding H.P., Ron D. (2001). Feedback inhibition of the unfolded protein response by GADD34-mediated dephosphorylation of eIF2 alpha. J. Cell Biol..

[bib20] Rivera J., Abrams C., Hernaez B., Alcazar A., Escribano J.M., Dixon L., Alonso C. (2007). The MyD116 African swine fever virus homologue interacts with the catalytic subunit of protein phosphatase 1 and activates its phosphatase activity. J. Virol..

[bib21] Rojas M., Vasconcelos G., Dever T.E. (2015). An eIF2 alpha-binding motif in protein phosphatase 1 subunit GADD34 and its viral orthologs is required to promote dephosphorylation of eIF2 alpha. Proc. Natl. Acad. Sci. USA.

[bib22] Urano F., Wang X.Z., Bertolotti A., Zhang Y.H., Chung P., Harding H.P., Ron D. (2000). Coupling of stress in the ER to activation of JNK protein kinases by transmembrane protein kinase IRE1. Science.

[bib23] Zhang C., Tang J., Xie J., Zhang H., Li Y., Zhang J., Verpooten D., He B., Cao Y. (2008). A conserved domain of herpes simplex virus ICP340.5 regulates protein phosphatase complex in mammalian cells. Febs Lett..

[bib24] Zhang F., Moon A., Childs K., Goodbourn S., Dixon L.K. (2010). The African swine fever virus DP71L protein recruits the protein phosphatase 1 catalytic subunit To dephosphorylate eIF2 alpha and inhibits CHOP induction but Is dispensable for These activities during virus infection. J. Virol..

